# The Impact of Accelerated HF-rTMS on Canine Brain Metabolism: An [^18^F]-FDG PET Study in Healthy Beagles

**DOI:** 10.3389/fvets.2022.800158

**Published:** 2022-02-24

**Authors:** Yangfeng Xu, Kathelijne Peremans, Jan Courtyn, Kurt Audenaert, Andre Dobbeleir, Yves D'Asseler, Eric Achten, Jimmy Saunders, Chris Baeken

**Affiliations:** ^1^Ghent Experimental Psychiatry (GHEP) Laboratory, Department of Head and Skin, Faculty of Medicine and Health Sciences, Ghent University, Ghent, Belgium; ^2^Department of Veterinary Medical Imaging and Small Animal Orthopaedics, Faculty of Veterinary Medicine, Ghent University, Merelbeke, Belgium; ^3^Department of Radiology and Nuclear Medicine, Medical Molecular Imaging and Therapy, Ghent University Hospital, Ghent, Belgium; ^4^Department of Head and Skin, Faculty of Medicine and Health Sciences, Ghent University Hospital, Ghent University, Ghent, Belgium; ^5^Department of Psychiatry, Faculty of Medicine and Pharmacy, Vrije University Brussels, Brussels, Belgium; ^6^Department of Electrical Engineering, Eindhoven University of Technology, Eindhoven, Netherlands

**Keywords:** aHF-rTMS, canine model, [^18^F]-FDG brain imaging, repetitive transcranial magnetic stimulation, neuropsychiatric disorders (NPD)

## Abstract

**Background:**

Repetitive transcranial magnetic stimulation (rTMS) has been proven to be a useful tool for the treatment of several severe neuropsychiatric disorders. Accelerated (a)rTMS protocols may have the potential to result in faster clinical improvements, but the effects of such accelerated paradigms on brain function remain to be elucidated.

**Objectives:**

This sham-controlled arTMS study aimed to evaluate the immediate and delayed effects of accelerated high frequency rTMS (aHF-rTMS) on glucose metabolism in healthy beagle dogs when applied over the left frontal cortex.

**Methods:**

Twenty-four dogs were randomly divided into four unequal groups: five active (*n* = 8)/ sham (*n* = 4) stimulation sessions (five sessions in 1 day), 20 active (*n* = 8)/ sham (*n* = 4) stimulation sessions (five sessions/ day for 4 days), respectively. [^18^F] FDG PET scans were obtained at baseline, 24 h poststimulation, after 1 and 3 months post the last stimulation session. We explicitly focused on four predefined regions of interest (left/right prefrontal cortex and left/right hippocampus).

**Results:**

One day of active aHF-rTMS- and not sham- significantly increased glucose metabolism 24 h post-active stimulation in the left frontal cortex only. Four days of active aHF-rTMS only resulted in a nearly significant metabolic decrease in the left hippocampus after 1 month.

**Conclusions:**

Like in human psychiatric disorders, active aHF-rTMS in healthy beagles modifies glucose metabolism, although differently immediately or after 1 month post stimulation. aHF-rTMS may be also a valid option to treat mentally disordered dogs.

## Introduction

Repetitive transcranial magnetic stimulation (rTMS) is an FDA-approved, non-invasive neurostimulation technique, which has been widely used in human medicine for major depressive disorder, anxiety, and epilepsy (1–4), as well as in veterinary medicine for epilepsy and anxiety in dogs (5–7). However, rTMS parameters still need to be optimized, and the underlying neurobiological mechanisms of action are not yet unraveled.

Positron emission tomography (PET) and single photo emission tomography (SPECT) have been extensively used in neuroscience research comaring humane and canine species, and several similarities were found in neurological and neuropsychiatric diseases like impulsive aggression, anxiety, and compulsive disorders (8–12). In our former research, we have already proposed that dogs can be a robustly translational animal model for human medicine (6, 13). According to this previous research using Hexamethylpropyleneamine oxime-SPECT (HMPAO-SPECT), high-frequency repetitive transcranial magnetic stimulation (HF-rTMS) applied to the left frontal cortex alters regional cerebral perfusion in healthy dogs (14). Moreover, measured with 3-amino-4-(2-dimethylaminomethylphenylsulfanyl)-benzonitrile-PET ([^11^C] DASB-PET), serotonin transporter (SERT) binding index alterations in different brain regions were reported (15). Similar changes have been observed in humans (1, 16). This strengthens the idea that further functional imaging techniques in dogs could not only be used to investigate the underlying neurobiological mechanisms, but it can also be used for the refinement of more effective rTMS treatment parameters in men and dogs.

One brain imaging method in particular, 2-deoxy-2-[^18^F] fluoro-D-glucose ([^18^F]-FDG), may prove also to be very informative. It reflects the distribution of glucose uptake and phosphorylation by cells. Most of the ATP required for brain function is from glucose metabolism, which is tightly connected to neuronal activity (17). It has potential use for antemortem diagnosis without histologic analysis and for monitoring response to treatment (18). The alteration in glucose metabolism therefore represents a change of neuronal activity induced by the disease (17, 19, 20) and the effects of treatment paradigms (21–23). In a previous study by our group, ^18^FDG-PET findings in melancholic refractory major depressive disorder (MDD) patients indicated that higher baseline subgenual anterior cingulate cortex (sgACC) metabolic activity may predict clinical outcome with repetitive accelerated (a)HF-rTMS treatment (21). According to previous studies, [^18^F]-FDG could be used in dogs for cerebral physiologic and pathophysiologic metabolism measurements (11, 24, 25).

This study aimed to investigate the effect of 1-day active/sham and 4-day active/sham aHF-rTMS on cerebral glucose metabolism, as assessed by [^18^F]-FDG in healthy beagles. Because the neurobiological effects in humans of accelerated rTMS paradigms have been found to affect prefrontal cortical and hippocampal areas (26–29), we explicitly focused on these regions. We hypothesized that active aHF-rTMS and not sham would alter the cerebral glucose metabolism in these regions at different time points. We also hypothesized that the effects of 1 day active (five stimulation sessions) and a complete active aHF-rTMS treatment protocol (20 sessions) would results in different metabolic effects.

## Materials and Methods

### Animals

**Figure 1:**
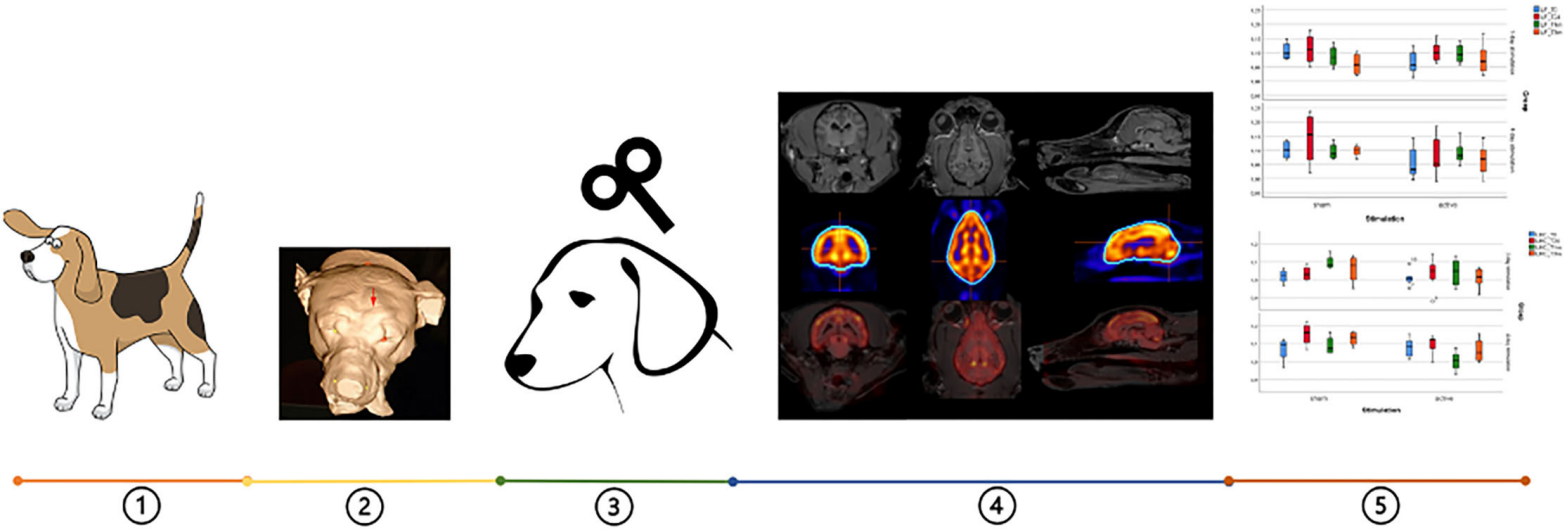
Pipeline of the whole experimental protocol: (1) twelve beagles were used, (2) neuronavigation was conducted to locate the accurate TMS point (red arrow), (3) four types of aHF-rTMS protocols were applied, (4) Images with different regions on the transversal, sagittal and dorsal slices: MRI, [^18^ F]-FDG PET and the fusion of both images, (5) data analysis.

The pipeline of the whole experimental protocol is shown in Figure 1. Twelve healthy beagle dogs (seven females neutered, five males castrated) were used in this study. Of note, all dogs were reused after a 3-month wash-out period (3 months after the last stimulation session) and with a confirmed return to glucose metabolism baseline checked by an [^18^F]-FDG PET scan. These dogs were owned by the Ghent University Department of Small Animals and the Department of Veterinary Medical Imaging and Small Animal Orthopedics. They were housed in groups of eight on an internal surface of 15 m^2^, with access to an outside area of 15 m^2^. The floor covering in the inner part consisted of wood shavings. Toys were given to these dogs every day and they were released to an enclosed playground twice a day. In addition, the veterinary students and animal house managers walked the dogs regularly. By using positive reinforcement techniques, all dogs were accustomed to the researchers, stimulating room and sound and placing of a sham coil. This was done several months before the start of the stimulation experiment. All dogs displayed normal behavior, regularly evaluated by both veterinarians involved and care takers of the dogs. Behavior remained impeccable over the whole study period. And the beagles didn't show any behavioral abnormalities after TMS treatment. The guidelines for animal welfare imposed by the ethical committee were respected. This study (EC numbers: 2018-09, 2018-088) was approved by the Ghent University Ethical Committee.

### Imaging Procedure

All dogs were scanned on a Biograph mCT 20 imaging system (Siemens, Knoxville, USA) with a 78 cm wide bore, LSO crystals and a true V option extending the field of view to 21.6 cm. Prior to the PET scan, a low dose CT survey (120 kV, 35 mA, pitch of 0.7, 20 slices of 3 mm) was conducted and used for attenuation correction and anatomical framework. A static PET scan of 10 min was performed 30 min after tracer administration (intravenous injection of 148 MBq [^18^F]-FDG). Emission data were corrected for dead time, scatter and random events, and reconstructed in a 512 × 512 matrix with a voxel size of 0.797 × 0.797 × 2 mm, using the TrueX algorithm.

### Neuronavigation

The aHF-rTMS treatment was applied over the left frontal cortex in this study. For neuronavigation purposes, each dog underwent a magnetic resonance imaging (MRI) scan in a Siemens 3T Magnetom Trio Tim system (Siemens Medical Systems, Erlangen, Germany) with the following sequence parameters: a MP-RAGE sequence with the following parameters: repetition time (TR) = 2,250 ms, echo time (TE) = 4.18 ms, matrix size = 256 × 256, field of view (FOV) = 256 × 256 mm^2^, flip angle = 9°, and voxel size=1 × 1 × 1 mm^3^, 176 slices. Then, a frameless neuronavigation system was used to provide the external localization of the left frontal cortex of every dog as described by Dockx et al. (14).

### The aHF-rTMS Protocol

The aHF-rTMS protocol was chosen according the treatment for human patients (21, 30) and evidence-based guidelines (31, 32). All stimulations were applied under general anesthesia. As performed by Dockx et al. (33), premedication consisted of butorphanol IV (0.2 mg/kg; Dolorex1; Intervet Belgium NV). After the onset of sedation, anesthesia was induced intravenously by administering midazolam (0.2 mg/kg; Dormicum; Roche Nederland B.V.), followed by propofol (Propovet Multidose, Abbott Laboratories, Berkshire, UK, 1 ± 2 mg/kg given to effect). General anesthesia was maintained with isoflurane (Isoflo, Abbott Laboratories, Berkshire, UK) in oxygen using a rebreathing system. Following the induction of general anesthesia, for each dog, the motor threshold of the left motor cortex was determined. A motor threshold (MT) of 100% was defined as the set machine output (Magstim Company Limited, Minneapolis, USA) that could provoke five out of 10 visible muscle contractions in the right upper front limb. After the assessment of the MT, the external localization of the center of the left frontal cortex was located based on the previously measured X, Y positions and marked with a permanent marker on the fur. The center of a human figure-of-eight coil was placed perpendicular over the mark with the handle pointing abaxial. For the sham group, the coil was placed at a 90-degree angle with one wing contacting the skull. HF-rTMS (20 Hz, 110% MT) was applied to the left frontal cortex. Each session contained 40 trains of 1.9 s each. The trains were separated by a 12 s intertrain interval (in total 1,560 pulses were given per session).

In the first experimental part, dogs were randomized to receive accelerated active or sham HF-rTMS (ratio 2:1) where five sessions were applied in a single day (1-day stimulation protocol). The time interval between sessions was 10 to 15 min. Scanning was performed before stimulation (T_0_ baseline), 24 h following the experiment (T_1_), and 1 month later to detect delayed effects (T_2_). An additional PET scan was performed 3 months after the experimental protocol to verify the normalization of metabolic brain patterns (T_3_).

In the second experimental part, after an extra period of 3 months, the dogs were reused. The same cohort of dogs was randomized again to receive 20 HF-rTMS sessions (ratio 2:1) of active or sham stimulation (five daily sessions over 4 days of stimulation) (4-day stimulation protocol). The time interval between sessions was again 10 to 15 min. Like the 1-day stimulation, [^18^F]-FDG PET scans were performed before stimulation (T_0_ baseline), 24 h following the experiment (T_1_), and 1 month later to detect delayed effects (T_2_). Again, an additional PET scan was performed 3 months after the experimental protocol to verify the normalization of metabolic brain patterns (T_3_).

### PET Analysis

[^18^F]-FDG PET images of each dog were automatically registered to a template generated from 14 beagle dogs (nine males, five females, mean age 50 ± 20 months), using Brain Registration and Automated SPECT Semiquantification (BRASS, Nuclear Diagnostics) software (34). This template-based automated registration method eliminates the subjective operator-dependent region definition and facilitates the fitting procedure. The latter is necessary to compensate for intra-individual differences in anatomical brain size and shape. According to a canine brain atlas (35, 36), a region map was generated on this template, including 21 separated manually drawn volumes of interest (VOI), positioned over the frontal, temporal, parietal, and occipital lobes of both hemispheres, and over the cerebellum and subcortical structures. In this study, regional activity within a predefined VOI was calculated by normalizing the regional registered activity to total brain activity.

### Statistical Analysis

Given the relatively small sample size, we applied only nonparametric analyses. According to a canine brain atlas (35, 36), we selected four regions of interest: the left and right prefrontal cortex and the left and right hippocampus. We analyzed the effects of the 1-day stimulation and 4-day stimulation on three time points (T_0_, T_1_, T_2_) separately. The last scan after three months (T_3_) was used to evaluate the normalization of brain metabolism patterns in the selected regions of interest. Because we hypothesized that active aHF-rTMS and not sham would impact glucose metabolism in the four selected regions of interest at one of the two time points following stimulation (T_1_, T_2_), Friedman's ANOVAs were Bonferroni corrected for four measurements (*p* < 0.0125, one-tailed). Significant outcomes were followed by Mann-Whitney U and Wilcoxon signed-rank tests, Bonferroni corrected for three time points (T_0_, T_1_, T_2_). For these *post hoc* analyses, significance was set at *p* < 0.0167, two-tailed. All other relevant statistical analyses significance was set at *p* < 0.05, two-tailed.

## Results

**Figure 2:**
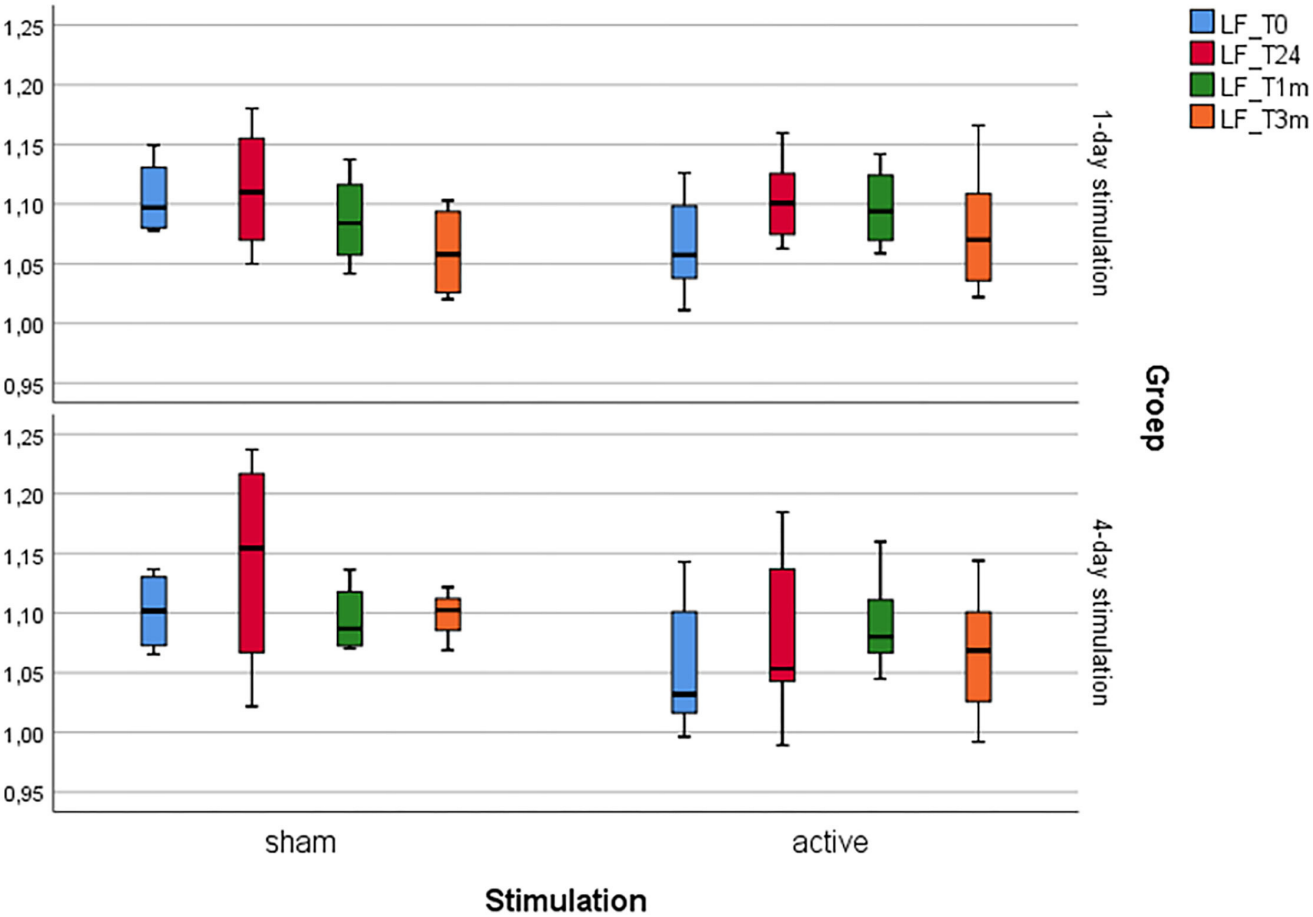
Boxplot showing the glucose metabolism changes of the left prefrontal cortex.

**Figure 3:**
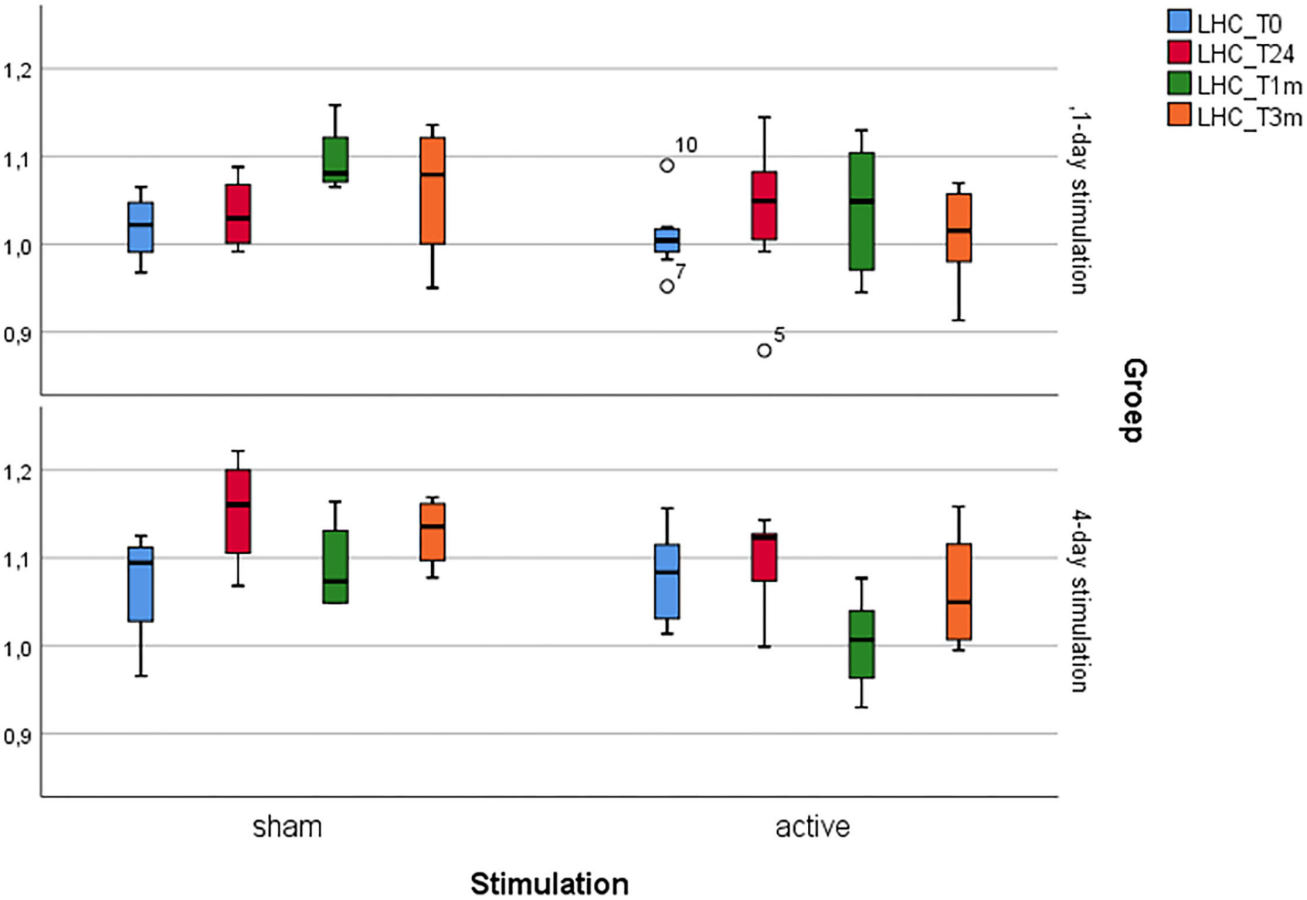
Boxplot showing the glucose metabolism changes of the left hippocampus.

For an overview (see Table 1 and Figures 2, 3).

**Table 1:** Statistical results of the different aHF-aTMS protocols.

***AHF-rTMS protocol***			**T0**	**T1**	**T2**	**T3**		**T0**	**T1**	**T2**	**T3**
*1-day stimulation*	Active						Sham				
		Left prefrontal cortex	1.06 (0.12)	1.10 (0.10)	1.09 (0.08)	1.07 (0.14)		1.10 (0.07)	1.11 (0.13)	1.08 (0.10)	1.06 (0.08)
		Right prefrontal cortex	1.08 (0.11)	1.09 (0.08)	1.11 (0.15)	1.09 (0.11)		1.12 (0.15)	1.11 (0.07)	1.08 (0.14)	1.10 (0.11)
		Left hippocampus	1.00 (0.14)	1.05 (0.27)	1.05 (0.18)	1.02 (0.16)		1.02 (0.10)	1.03 (0.10)	1.08 (0.09)	1.08 (0.19)
		Right hippocampus	0.99 (0.12)	1.04 (0.19)	1.04 (0.13)	0.10 (0.20)		0.98 (0.11)	0.98 (0.08)	1.06 (0.09)	0.98 (0.16)
*4-day stimulation*	Active						Sham				
		Left prefrontal cortex	1.03 (0.15)	1.05 (0.20)	1.08 (0.11)	1.07 (0.15)		1.10 (0.07)	1.15 (0.22)	1.09 (0.07)	1.10 (0.05)
		Right prefrontal cortex	1.14 (0.24)	1.07 (0.12)	1.09 (0.12)	1.10 (0.16)		1.11 (0.13)	1.12 (0.13)	1.11 (0.08)	1.09 (0.08)
		Left hippocampus	1.08 (0.14)	1.12 (0.14)	1.01 (0.15)	1.05 (0.16)		1.09 (0.16)	1.16 (0.15)	1.07 (0.12)	1.14 (0.09)
		Right hippocampus	1.05 (0.23)	1.07 (0.20)	0.99 (0.15)	1.03 (0.21)		1.04 (0.12)	1.03 (0.21)	1.03 (0.10)	1.05 (0.12)

### One-Day Stimulation

First, Mann-Whitney U tests did not show any significantly different baseline glucose metabolism patterns in any of the four regions of interest before 1 day of active and sham aHF-rTMS (*p's* > 0.05).

For the right prefrontal cortex and the left and right hippocampus, Friedman's ANOVAs showed no significance in metabolic changes for active and sham aHF-rTMS in any of the three timepoints (T_0_, T_1_, T_2_), (*p's* > 0.05).

Friedman's ANOVA for the left prefrontal cortex showed a significant difference between the three time points after active aHF-rTMs (*X*^2^(2) = 9.00, *p* = 0.010), not after sham (*X*^2^(2) = 2.00, *p* = 0.431). Wilcoxon signed-rank *post hoc* test revealed a significant increase of glucose metabolism 24 h post-active stimulation (*z* = −2.521, *n*(ties) = 8, *p* = 0.012, *r* = −0.63, 24 h post). Mann-Whitney U tests did not show any significant different glucose metabolism between sham and active aHF-rTMS at any of the poststimulation time points (T_1_, T_2_) (*p's* > 0.05).

Finally, Mann-Whitney U and Wilcoxon signed-rank tests yielded no significant changes in brain glucose metabolism in the four predefined VOIs after 3 months of stimulation (T_3_), when compared to the baseline (T_0_). This indicates that 1 day of accelerated HF-rTMS in healthy dogs does not alter brain glucose metabolism in the long term.

### Four-Day Stimulation

Again, Mann-Whitney U tests did not show significantly different baseline glucose metabolism patterns in any of the four regions of interest before 4 days of active and sham aHF-rTMS (*p's* > 0.05).

Although Friedman's ANOVAs for the left and right prefrontal cortex and the right hippocampus showed no significant changes in glucose metabolism for active and sham aHF-rTMS in any of the three timepoints (*p's* > 0.05), Friedman's ANOVA for the left hippocampus showed a nearly significant difference after active aHF-rTMs (*X*^2^(2) = 7.00, *p* = 0.015) and not after sham (*X*^2^(2) = 1.500, *p* = 0.653). Given our a priori hypothesis that active and not sham aHF-rTMS would affect left hippocampal areas specifically, we further explored with the Wilcoxon signed-rank *post hoc* test, which revealed a near significant decrease of glucose metabolism 1 month poststimulation (*z* = −2.380, *n*(ties) = 8, *p* = 0.017, *r* = −0.60, 1 month post). Mann Whitney U tests showed near significant lower glucose metabolism 1 month post stimulation in the active condition when compared to sham (U = 2.00, n1 = 8, n2 = 4, *p* = 0.017, *r* = −0.69).

Finally, also after the 4-day aHF-rTMS protocol, Mann-Whitney U and Wilcoxon signed-rank tests yielded no significant changes in brain glucose metabolism in the four predefined regions of interest after 3 months of stimulation (T_3_), when compared to the baseline (T_0_). This indicates that also 4 days of accelerated HF-rTMS in healthy dogs does not alter brain glucose metabolism in the long term.

## Discussion

The results of this study indicate that active aHF-rTMS, 1-day or 4-days, applied to the left frontal cortex impact the cerebral glucose metabolism in different regions over time.

First, 1 day of active and not sham aHF-rTMS significantly increased glucose metabolism 24 h postactive stimulation only in the left prefrontal cortex. This was a relatively strong effect. This metabolic increase is of course not surprising, given that this was the stimulated area. Furthermore, the frontal cortex has been shown to be implicated in general anxiety disorder (GAD) and major depression disorder (MDD) in humans (37). MDD patients typically show a lower resting-state cerebral glucose uptake index in the left lateral orbitofrontal cortex, left/right dorsolateral prefrontal cortex, and left ventrolateral prefrontal cortex when compared to healthy volunteers (38, 39). In dogs with anxiety disorders, decreased perfusion was reported in the left frontal cortex (10). Furthermore, animal research indicates that anxiety elicits a sustained background “hypofrontality” in the dorsomedial prefrontal cortex (dmPFC) due to a reduced recruitment of dmPFC neurons, reducing the firing rate of spontaneously active neuronal subpopulations (40). Because left frontal cortical metabolism has often been found to be decreased in major depression, one might exp ect fast left frontal rTMS to “normalize” metabolism and thus be more effective than slow left frontal rTMS in improving mood (41). Indeed, also in human research 20 Hz rTMS delivered over the left prefrontal cortex was associated with significant increases in regional cerebral blood flow (rCBF) across the group of all 10 MDD patients, which were located in the prefrontal cortex (L > R) (42). On the other hand, we did not observe a similar increase after the 4-day stimulation protocol. This suggests that 1 and 4 days of accelerated HF-rTMS does not automatically mean higher neuronal activity resulting from multiple stimulation days in the stimulated area. It also suggests no carry-over effects from the first 1-day stimulation to the 4-days stimulation, so many months later. At this point, however, it is speculative to assume that habituation could have occurred by stimulating daily, and that the prefrontal cortex strives to “normalization” the regional metabolic activity. It can also just indicate that in healthy beagle dogs brain glucose metabolism returns relatively fast to baseline.

Second, although we observed nearly significant decreases in left hippocampal brain glucose metabolism 1 month post active 4-day stimulation, the effect size of this decrease was also quite high. Delayed biological effects in the (left) hippocampus after rTMS in humans as well as in animal studies have been reported. Although the interpretation here should be done with caution, given that these were healthy beagle dogs; these observations indicate some form of delayed effect on neuronal activity after prolonged accelerated stimulation. However, to provide a potential biological explanation, it must be mentioned that functional and structural connections between the prefrontal cortex and the hippocampus exist, playing an important role in cognitive functioning and a disrupted interaction may contribute to the pathophysiology of various psychiatric diseases such as MDD (43, 44). It was reported that the structure-function relationships in rTMS-induced neuroplastic changes were mediated through the hippocampus and prefrontal network at the stimulated side, and high-frequency prefrontal rTMS may exert its cognitive effects through the hippocampal structural functional neuroplasticity (36). Importantly, in this study, the effects of aHF-rTMS were both found in the left side, suggesting structural connections between the prefrontal cortex and hippocampus. In a recent accelerated intermittent theta burst stimulation study in human MDD, we also found that active and not sham accelerated stimulation resulted in structural changes in the left dentate gyrus and part of the hippocampus, and in relation to clinical improvement (45). Although these were healthy beagles, it is tempting to assume that the prolonged effect of aHF-rTMS on left hippocampal metabolism are related to the neurobiological effects of this kind of stimulation. Nevertheless, this change in left hippocampal metabolic activity normalized 3 months later. This indicates that in healthy dogs, 4 days of aHF-rTMS does not alter brain glucose metabolism in the long run. On the other hand, not continuing treatment after successful rTMS in MDD may result in high relapse rates within 1 year post stimulation (46). Our findings support the assumption that at least the rTMS treatment effects have to be considered as temporary, and post-treatment sessions, such as maintenance rTMS, may be mandatory to sustain the antidepressant effect (47).

Some limitations must be mentioned. First, this is a single blinded study, the handler is not blinded. At this point, we can only report these changes without associating this with behavioral effects, because we did not include behavioral assessments here, and the beagle dogs had no diagnosis of pathological behavior. Secondly, the aHF-rTMS protocol was only applied in a limited number of healthy dogs, due to our ethical restrictions, where all of them were reused for stimulation in line with the triple R (replace, reduce and refine) rules imposed by ethical regulations for experimental animals. Thirdly, during the sham treatment, an active coil was placed over the left frontal cortex, tilted 90 degrees. An active coil placed in this manner could provoke minor voltages in the underlying cortical tissue (48). Lastly, our protocol was performed under anesthesia. Although we used the same anesthetic protocol for all procedures, this might cause deviations from the awake condition.

## Conclusion

Our [^18^F]-FDG study in healthy beagle dogs showed immediate and delayed effects of aHF-rTMS differently in the left prefrontal cortex and the left hippocampus. The short-term left prefrontal metabolic increase may indicate the start of the metabolic cascade toward the subcortical connected brain regions. This delayed left hippocampal decrease may point to the presumably therapeutic effect as this is in line with some other antidepressant treatments, where meaningful clinical effects are to be expected after a couple of weeks and not immediately. In summary, this study provides more insights into the mechanism of aHF-rTMS in dogs, benefiting both veterinary medicine and human medicine. Of course, it remains to be determined whether accelerated low frequency rTMS would yield similar metabolic changes in these kinds of healthy dogs.

## Data Availability Statement

The original contributions presented in the study are included in the article/supplementary material, further inquiries can be directed to the corresponding author.

## Ethics Statement

The animal study was reviewed and approved by Ghent University Ethics Committee.

## Author Contributions

YX: data curation, formal analysis, investigation, methodology, software, visualization, and writing–original draft. KP and CB: conceptualization, project administration, resources, formal analysis, supervision, and writing–review and editing. JC, KA, and JS: conceptualization, resources, supervision, and writing–review and editing. AD, YD'A, and EA: conceptualization, resources, and writing–review and editing. All authors contributed to the article and approved the submitted version.

## Funding

This study is funded by Belgium Governmental FWO Institution (Project number G011018N). This research was supported by FWO grant G011018N.

## Conflict of Interest

The authors declare that the research was conducted in the absence of any commercial or financial relationships that could be construed as a potential conflict of interest.

## Publisher's Note

All claims expressed in this article are solely those of the authors and do not necessarily represent those of their affiliated organizations, or those of the publisher, the editors and the reviewers. Any product that may be evaluated in this article, or claim that may be made by its manufacturer, is not guaranteed or endorsed by the publisher.

## References

[1] Baeken C Marinazzo D Wu G-R Van Schuerbeek P De Mey J Marchetti I. Accelerated HF-rTMS in treatment-resistant unipolar depression: Insights from subgenual anterior cingulate functional connectivity. World J Biol Psychiatry. (2014) 15:286–97. 10.3109/15622975.2013.872295 24447053

[2] George MS Nahas Z Molloy M Speer AM Oliver NC Li X-B. A controlled trial of daily left prefrontal cortex TMS for treating depression. Biol Psychiatry. (2000) 48:962–70. 10.1016/S0006-3223(00)01048-9 11082469

[3] Manganotti P Tamburin S Zanette G Fiaschi A. Hyperexcitable cortical responses in progressive myoclonic epilepsy: a TMS study. Neurology. (2001) 57:1793–9. 10.1212/WNL.57.10.1793 11723265

[4] Horvath JC Mathews J Demitrack MA Pascual-Leone A. The NeuroStar TMS device: conducting the FDA approved protocol for treatment of depression. JoVE. (2010) 45:e2345. 10.3791/2345 21189465 PMC3159591

[5] Nollet H Van Ham L Deprez P Vanderstraeten G. Transcranial magnetic stimulation: review of the technique, basic principles and applications. Vet J. (2003) 166:28–42. 10.1016/S1090-0233(03)00025-X 12788015

[6] Dockx R Baeken C Duprat R De Vos F Saunders J Polis I. Changes in canine cerebral perfusion after accelerated high frequency repetitive transcranial magnetic stimulation (HF-rTMS): a proof of concept study. Vet J. (2018) 234:66–71. 10.1016/j.tvjl.2018.02.004 29680396

[7] Charalambous M Van Ham L Broeckx BJ Roggeman T Carrette S Vonck K. Repetitive transcranial magnetic stimulation in drug-resistant idiopathic epilepsy of dogs: a noninvasive neurostimulation technique. J Vet Intern Med. (2020) 34:2555–61. 10.1111/jvim.15919 33009717 PMC7694858

[8] Audenaert K Van Laere K Dumont F Slegers G Mertens J van Heeringen C. Decreased frontal serotonin 5-HT 2a receptor binding index in deliberate self-harm patients. Eur J Nucl Med. (2001) 28:175–82. 10.1007/s002590000392 11303887

[9] Eren I Tükel R Polat A Karaman R Ünal S. Evaluation of regional cerebral blood flow changes in panic disorder with Tc99m-HMPAO SPECT. Psychiatry Res. (2003) 123:135–43. 10.1016/S0925-4927(03)00062-3 12850252

[10] Vermeire S Audenaert K Dobbeleir A De Meester R Vandermeulen E Waelbers T. Regional cerebral blood flow changes in dogs with anxiety disorders, measured with SPECT. Brain Imaging Behav. (2009) 3:342–9. 10.1007/s11682-009-9076-1

[11] Irimajiri M Miller MA Green MA Jaeger CB Luescher AU Hutchins GD. Cerebral metabolism in dogs assessed by 18F-FDG PET: a pilot study to understand physiological changes in behavioral disorders in dogs. J Vet Med Sci. (2010) 72:1–6. 10.1292/jvms.09-0048 19861888

[12] Zitterl W Aigner M Stompe T Zitterl-Eglseer K Gutierrez-Lobos K Wenzel T. Changes in thalamus–hypothalamus serotonin transporter availability during clomipramine administration in patients with obsessive–compulsive disorder. Neuropsychopharmacology. (2008) 33:3126–34. 10.1038/npp.2008.35 18354388

[13] Dockx R Baeken C De Bundel D Saunders J Van Eeckhaut A Peremans K. Accelerated high-frequency repetitive transcranial magnetic stimulation positively influences the behavior, monoaminergic system, and cerebral perfusion in anxious aggressive dogs: A case study. J Vet Behav. (2019) 33:108–13. 10.1016/j.jveb.2019.07.004

[14] Dockx R Peremans K Duprat R Vlerick L Van Laeken N Saunders JH. Accurate external localization of the left frontal cortex in dogs by using pointer based frameless neuronavigation. PeerJ. (2017) 5:e3425. 10.7717/peerj.3425 28713649 PMC5507169

[15] Dockx R. The human dog: a translational neurobiological brain model on the molecular effects of the non-invasive brain stimulation technique accelerated high frequency repetitive transcranial magnetic stimulation (HF-rTMS). Ghent: Ghent University (2019).

[16] Speer A Benson B Kimbrell T Wassermann E Willis M Herscovitch P. Opposite effects of high and low frequency rTMS on mood in depressed patients: relationship to baseline cerebral activity on PET. J Affect Disord. (2009) 115:386–94. 10.1016/j.jad.2008.10.006 19027962 PMC2779113

[17] Varrone A Asenbaum S Vander Borght T Booij J Nobili F Någren K. EANM procedure guidelines for PET brain imaging using [18 F] FDG, version 2. Eur J Nucl Med Mol Imaging. (2009) 36:2103–10. 10.1007/s00259-009-1264-0 19838705

[18] Williams LM Morandi F Osborne DR Narak J LeBlanc AK. Kinetic analysis of 2-([18F]fluoro)-2-deoxy-d-glucose uptake in brains of anesthetized healthy dogs. Am J Vet Res. (2014) 75:588–94. 10.2460/ajvr.75.6.588 24866515

[19] Chen W Cloughesy T Kamdar N Satyamurthy N Bergsneider M Liau L. Imaging proliferation in brain tumors with 18F-FLT PET: comparison with 18F-FDG. J Nucl Med. (2005) 46:945–52. 15937304

[20] Li YQ Liao XX Lu JH Liu R Hu CL Dai G. Assessing the early changes of cerebral glucose metabolism *via* dynamic (18)FDG-PET/CT during cardiac arrest. Metab Brain Dis. (2015) 30:969–77. 10.1007/s11011-015-9658-0 25703241

[21] Baeken C Marinazzo D Everaert H Wu G-R Van Hove C Audenaert K. The impact of accelerated HF-rTMS on the subgenual anterior cingulate cortex in refractory unipolar major depression: insights from 18FDG PET brain imaging. Brain Stimul. (2015) 8:808–15. 10.1016/j.brs.2015.01.415 25744500

[22] Ketter TA Kimbrell TA George MS Dunn RT Speer AM Benson BE. Effects of mood and subtype on cerebral glucose metabolism in treatment-resistant bipolar disorder. Biol Psychiatry. (2001) 49:97–109. 10.1016/S0006-3223(00)00975-6 11164756

[23] Videbech P. PET measurements of brain glucose metabolism and blood flow in major depressive disorder: a critical review. Acta Psychiatr Scand. (2000) 101:11–20. 10.1034/j.1600-0447.2000.101001011.x 10674946

[24] Kang B-T Son Y-D Lee S-R Jung D-I Kim D-E Chang K-T. FDG uptake of normal canine brain assessed by high-resolution research tomography-positron emission tomography and 7 T-magnetic resonance imaging. J Vet Med Sci. (2012) 74:1261–7. 10.1292/jvms.12-0107 22673724

[25] Jokinen TS Haaparanta-Solin M Viitmaa R Grönroos TJ Johansson J Bergamasco L. FDG-Pet in healthy and epileptic lagotto romagnolo dogs and changes in brain glucose uptake with age. Vet Radiol Ultrasound. (2014) 55:331–41. 10.1111/vru.12129 24354474

[26] Kreuzer PM Downar J de Ridder D Schwarzbach J Schecklmann M Langguth B. Comprehensive review of dorsomedial prefrontal cortex rTMS utilizing a double cone coil. Neuromodulation. (2019) 22:851–66. 10.1111/ner.12874 30411429

[27] Freedberg M Reeves JA Toader AC Hermiller MS Voss JL Wassermann EM. Persistent enhancement of hippocampal network connectivity by parietal rTMS is reproducible. eNeuro. (2019) 6:ENEURO.0129-19.2019. 10.1523/ENEURO.0129-19.2019 31591137 PMC6795558

[28] Ogiue-Ikeda M Kawato S Ueno S. The effect of repetitive transcranial magnetic stimulation on long-term potentiation in rat hippocampus depends on stimulus intensity. Brain Res. (2003) 993:222–6. 10.1016/j.brainres.2003.09.009 14642850

[29] Nahas Z Teneback CC Kozel A Speer AM DeBrux C Molloy M. Brain effects of TMS delivered over prefrontal cortex in depressed adults: role of stimulation frequency and coil–cortex distance. J Neuropsychiatry Clin Neurosci. (2001) 13:459–70. 10.1176/jnp.13.4.459 11748315

[30] Baeken C Vanderhasselt M-A Remue J Herremans S Vanderbruggen N Zeeuws D. Intensive HF-rTMS treatment in refractory medication-resistant unipolar depressed patients. J Affect Disord. (2013) 151:625–31. 10.1016/j.jad.2013.07.008 23896317

[31] Lefaucheur J-P André-Obadia N Antal A Ayache SS Baeken C Benninger DH. Evidence-based guidelines on the therapeutic use of repetitive transcranial magnetic stimulation (rTMS). Clin Neurophysiol. (2014) 125:2150–206. 10.1016/j.clinph.2014.05.021 25034472

[32] Lefaucheur J-P Aleman A Baeken C Benninger DH Brunelin J Di Lazzaro V. Evidence-based guidelines on the therapeutic use of repetitive transcranial magnetic stimulation (rTMS): an update (2014–2018). Clin Neurophysiol. (2020) 131:474–528. 10.1016/j.clinph.2019.11.002 31901449

[33] Dockx R Peremans K Vlerick L Van Laeken N Saunders JH Polis I. Anaesthesia, not number of sessions, influences the magnitude and duration of an aHF-rTMS in dogs. PLoS ONE. (2017) 12:e0185362. 10.1371/journal.pone.0185362 28937993 PMC5609759

[34] Peremans K Audenaert K Coopman F Blanckaert P Jacobs F Otte A. Estimates of regional cerebral blood flow and 5-HT2A receptor density in impulsive, aggressive dogs with 99m Tc-ECD and 123 I-5-I-R91150. Eur J Nucl Med Mol Imaging. (2003) 30:1538–46. 10.1007/s00259-003-1250-x 14579095

[35] Czeibert K Andics A Petneházy Ö Kubinyi E. A detailed canine brain label map for neuroimaging analysis. Biologia Futura. (2019) 70:112–20. 10.1556/019.70.2019.14 34554420

[36] Nitzsche B Boltze J Ludewig E Flegel T Schmidt MJ Seeger J. A stereotaxic breed-averaged, symmetric T2w canine brain atlas including detailed morphological and volumetrical data sets. Neuroimage. (2019) 187:93–103. 10.1016/j.neuroimage.2018.01.066 29407456

[37] Carlson JM Rubin D Mujica-Parodi LR. Lost emotion: disrupted brain-based tracking of dynamic affective episodes in anxiety and depression. Psychiatry Res Neuroimaging. (2017) 260:37–48. 10.1016/j.pscychresns.2016.12.002 28013067

[38] Martinot M-LP Martinot J-L Ringuenet D Galinowski A Gallarda T Bellivier F. Baseline brain metabolism in resistant depression and response to transcranial magnetic stimulation. Neuropsychopharmacology. (2011) 36:2710–9. 10.1038/npp.2011.161 21849980 PMC3230494

[39] Li C-T Wang S-J Hirvonen J Hsieh J-C Bai Y-M Hong C-J. Antidepressant mechanism of add-on repetitive transcranial magnetic stimulation in medication-resistant depression using cerebral glucose metabolism. J Affect Disord. (2010) 127:219–29. 10.1016/j.jad.2010.05.028 20598753

[40] Park J Wood J Bondi C Del Arco A Moghaddam B. Anxiety evokes hypofrontality and disrupts rule-relevant encoding by dorsomedial prefrontal cortex neurons. J Neurosci. (2016) 36:3322–35. 10.1523/JNEUROSCI.4250-15.2016 26985040 PMC4792942

[41] Rosenberg PB Mehndiratta RB Mehndiratta YP Wamer A Rosse RB Balish M. Repetitive transcranial magnetic stimulation treatment of comorbid posttraumatic stress disorder and major depression. J Neuropsychiatry Clin Neurosci. (2002) 14:270–6. 10.1176/jnp.14.3.270 12154150

[42] Speer AM Kimbrell TA Wassermann EM Repella JD Willis MW Herscovitch P. Opposite effects of high and low frequency rTMS on regional brain activity in depressed patients. Biol Psychiatry. (2000) 48:1133–41. 10.1016/S0006-3223(00)01065-9 11137053

[43] Godsil BP Kiss JP Spedding M Jay TM. The hippocampal-prefrontal pathway: the weak link in psychiatric disorders? European Neuropsychopharmacol. (2013) 23:1165–81. 10.1016/j.euroneuro.2012.10.018 23332457

[44] Uhlhaas PJ Singer W. Neuronal dynamics and neuropsychiatric disorders: toward a translational paradigm for dysfunctional large-scale networks. Neuron. (2012) 75:963–80. 10.1016/j.neuron.2012.09.004 22998866

[45] Baeken C Wu G Sackeim HA. Accelerated iTBS treatment applied to the left DLPFC in depressed patients results in a rapid volume increase in the left hippocampal dentate gyrus, not driven by brain perfusion. Brain Stimul. (2020) 13:1211–7. 10.1016/j.brs.2020.05.015 32512184

[46] Janicak PG Nahas Z Lisanby SH Solvason HB Sampson SM McDonald WM. Durability of clinical benefit with transcranial magnetic stimulation (TMS) in the treatment of pharmacoresistant major depression: assessment of relapse during a 6-month, multisite, open-label study. Brain Stimul. (2010) 3:187–99. 10.1016/j.brs.2010.07.003 20965447

[47] Baeken C Brem AK Arns M Brunoni AR Filipčić I Ganho-Ávila A. Repetitive transcranial magnetic stimulation treatment for depressive disorders: current knowledge and future directions. Curr Opin Psychiatry. (2019) 32:409–15. 10.1097/YCO.0000000000000533 31145145 PMC6688778

[48] Baldinger P Kranz GS Haeusler D Savli M Spies M Philippe C. Regional differences in SERT occupancy after acute and prolonged SSRI intake investigated by brain PET. Neuroimage. (2014) 88:252–62. 10.1016/j.neuroimage.2013.10.002 24121201

